# Tumour budding in gastric and colorectal cancers: A review

**DOI:** 10.1097/MD.0000000000042215

**Published:** 2025-08-01

**Authors:** Hao Zhang, Xianyu Meng, Dongyun Gou, Yu Huang, Haiyang Wang, Hongyan Li

**Affiliations:** a Department of Abdominal Surgery, The Second People’s Hospital of Hengshui City, Hengshui City, Hebei Province, China; b Department of Gastrointestinal Surgery, The Third Hospital of Hebei Medical University, Shijiazhuang City, Hebei Province, China; c The Medical Record Room of the Medical Affairs Department, The First Hospital of Hebei Medical University, Shijiazhuang City, Hebei Province, China; d Department of Vascular Surgery, The Third Hospital of Hebei Medical University, Shijiazhuang City, Hebei Province, China.

**Keywords:** gastric and colorectal cancers, tumour budding

## Abstract

Tumour budding (TB), characterized by the presence of individual or small clusters of tumor cells at the infiltrative margins of malignant tumors, represents a critical aspect of tumor metastasis and progression. This review synthesizes current research on TB in gastric and colorectal cancers, focusing on its occurrence, biological characteristics, and clinical significance. We discuss the methodologies used for observing and grading TB, its association with the aggressiveness of digestive tract tumors, and the underlying molecular mechanisms, particularly epithelial-mesenchymal transition. Additionally, we explore potential new therapeutic targets related to TB, such as long noncoding RNAs (lncRNAs), microRNAs (miRNAs), and exosomes. By summarizing these findings, we aim to provide insights into the role of TB in gastric and colorectal cancer progression and guide future research directions.

## 1. Introduction

Malignant tumors of the digestive tract are broadly defined as those arising within the continuous muscular, hollow organ extending from the mouth to the anal canal, encompassing the mouth, pharynx, esophagus, stomach, small intestine, colorectum, and anal canal. According to cancer statistics published by the China Cancer Center in 2022, the combined incidence rate of stomach, colorectal, and esophageal cancers in China has reached 26.01%, with a corresponding death rate of 28.08%.^[1]^ These figures underscore the growing concern surrounding malignant tumors of the digestive tract. A notable characteristic of tumor budding is its association with the development of distant metastases. The process of distant metastasis in gastric and colorectal cancers is intricate and intricately linked to numerous factors. Research has indicated that tumor budding provides cellular-level insights into the mechanisms underlying tumor metastasis, thereby emerging as a crucial aspect of this phenomenon.^[2]^ This paper aims to provide a comprehensive review of the current research status regarding the occurrence, biological characteristics, and metastasis of tumor budding in gastric and colorectal cancers.

## 2. Overview of tumor budding

### 2.1. Definition of tumor budding

The medical community currently lacks a unified definition of tumor budding. The concept was initially introduced by Japanese scientist Imai in the 1950s.^[3]^ Despite its early proposal, tumor budding has received limited attention from scholars in subsequent years. A more widely accepted definition of the concept emerged at the 2016 International Tumor Budding Consensus Conference (ITBCC).^[4]^ At this conference, it was proposed that tumor budding (TB) refers to the dispersed presence of individual tumor cells or small clusters comprising ≤ 4 tumor cells, located at the infiltrative margins of normal tissue adjacent to a malignant tumor. Furthermore, the conference delineated clusters containing 5 or more cells as hypo-differentiated clusters. Tumour cells forming clusters larger than 5 cells can arrange into more stable glandular structures or nests, which to some extent diminishes their metastatic potential. This distinction based on a threshold of 5 tumor cells is pivotal for differentiating between tumor budding and hypo-differentiated clusters.

Tumour budding (TB) is a dynamic process indicative of a tumor’s capacity to metastasize to distant sites. It represents a cytological manifestation of metastasis occurring at the microscopic level within the tumor. Furthermore, Wendy introduced the concept of “discontinuous spread,”^[5]^ which emphasizes that a tumor nodule located within 1 mm of the margin of a malignant tumor should not be classified as tumor budding, as it may be connected to the tumor tissue in different planes. Therefore, when examining sections of tumors for budding in future studies, it is crucial to consider this concept and refrain from classifying nodules within 1 mm of the tumor margin as tumor budding. Additionally, to ascertain whether a particular site exhibits tumor budding, it is advisable to examine 2 consecutive sets of adjacent pathology sections.

### 2.2. Observation of tumor budding

Currently, the most frequently employed method for observing tumor budding is hematoxylin and eosin (HE) staining.^[6]^ Under a light microscope, tumor budding can typically be distinctly observed as cells or clusters of cells exhibiting significant heterogeneity and eosinophilic staining with HE. These cells often display pleomorphic nuclei, which are deeply stained, contain enlarged nucleoli, and exhibit pronounced abnormal mitotic figures.^[7]^ An alternative observation method is immunohistochemical staining (IHC). This method is generally preferred when there is a robust inflammatory reaction at the leading edge of tumor tissue infiltration or when there is a substantial amount of tissue debris, as the inflammatory response can interfere with the observation of tumor budding under HE staining, and it can be challenging to clearly distinguish tumor budding from tissue debris with HE staining.^[8]^ Consequently, IHC offers certain advantages over HE staining, including better reproducibility.^[9]^ Leão and several other scholars have deemed IHC to be a superior assay compared to HE staining, noting that in recent years, an increasing number of scholars have adopted IHC staining in order to minimize errors in observing tumor budding. However, at present, HE staining is still more widely utilized by researchers in China. Therefore, combining both methods may help reduce errors in the interpretation of tumor budding.

### 2.3. Counting and grading of tumor budding

The field of counting and grading tumor budding has evolved over time. The earliest research in this area was conducted by Haes, who defined the grading of tumor budding into 4 categories: “none,” “few,” “moderate,” and “severe,” but he did not provide a specific method for counting and grading.^[10]^ Subsequently, a new grading method was proposed by pathologist Ueno.^[11,12]^ In this method, the areas with the densest tumor budding, known as hotspots, were first selected microscopically, and counts of tumor budding were performed in these areas. Ueno method included 2 grading approaches based on counts: 1 divided the number of tumor budding into 4 groups (<5, 5–9, 10–19, and ≥20 per 25 fields of view, which corresponds to a 0.385 mm² area), and the other divided the number into 2 groups (<10 and ≥10 per 25 fields of view) with a cutoff value of 10.

At the subsequent ITBCC in 2016,^[4]^ a novel approach based on Ueno counting method was presented. This approach also involved observations and counts in the hotspot area, but the grading criteria were revised. The classification of tumor budding into low, intermediate, and high levels (0–4, 5–9, and ≥10 per 20 fields of view, which corresponds to a 0.785 mm² area) was proposed. This protocol was gradually accepted as the current standard grading method for tumor budding. By following this standardized approach, researchers can more accurately assess and compare tumor budding across different studies and patients.

## 3. Tumours of the digestive tract and tumor budding

### 3.1. Tumour budding phenomenon in gastric cancer

Gastric cancer is one of the most common malignancies of the digestive tract, with adenocarcinoma being the predominant subtype. The Lauren classification system, introduced in 1965, remains widely utilized for the categorization of gastric cancer.^[13]^ According to this classification, gastric cancer can be divided into intestinal-type gastric cancer (IGC) and diffuse-type gastric cancer (DGC). Both subtypes reflect, to some extent, the biological characteristics and origin of gastric cancer. Intestinal-type gastric cancer is frequently associated with intestinal epithelial metaplasia of the stomach and is most prevalent in older men, particularly in the antrum.^[13]^ Studies have demonstrated that intestinal-type gastric cancers are predominantly linked to H. pylori infection and generally exhibit a more favorable prognosis. In contrast, diffuse gastric cancer is more common in women and is prone to peritoneal metastases.^[13]^ This subtype of gastric cancer is characterized by intracellular mucus that can displace the nucleus, forming an imprint-like cell carcinoma, which typically has a poor prognosis. TB has been observed in various sites, including the lungs, breast, esophagus, stomach, and colorectum. Recent research has shown that tumor budding and lymphatic metastasis in a wide range of malignancies are strongly correlated with patient prognosis.^[2]^

As early as 2014, scholars such as Chen Junfang discussed the clinicopathological significance of tumor budding in digestive tract malignancies. However, subsequent research by domestic scholars on the relationship between gastric cancer and tumor budding has been relatively limited.^[14]^ Despite the limited focus on tumor budding in gastric cancer in recent years, its role in assessing metastasis and prognosis is consistent with that observed in other malignant tumors. For instance, Yin Kai study on nerve invasion and tumor budding in gastric cancer^[15]^ confirmed that gastric cancer cells and nerve cells exhibit a mutually promotional relationship. On 1 hand, neurofactors secreted by nerve cells can facilitate the metastasis, proliferation, and differentiation of tumor cells. On the other hand, tumor cells can also promote the growth of nerve cell axons in some manner. Furthermore, Yao et al investigated the relationship between tumor budding, lymphatic metastasis, and prognosis in early gastric cancer (EGC).^[16]^ They found that tumor budding was strongly associated with depth of infiltration, TNM stage, and lymphatic metastasis in EGC, independent of tumor size and histological type. Patients with high-grade tumor budding were more prone to lymphatic metastasis and had a poorer prognosis. In recent years, studies of tumor budding in gastric cancer have primarily focused on the intestinal type, with the prognosis of the diffuse type being worse compared to the intestinal type. Therefore, the current conclusions regarding tumor budding in gastric cancer may not be applicable to diffuse gastric cancer.^[17]^ For this reason, it is difficult to define tumor outgrowth from diffuse-type gastric cancer.^[18]^ Areas of isolated or small clusters of tumor cells are also considered to be diffuse-type gastric cancer.Tanaka as well as Stephen et al have excluded diffuse-type gastric cancer because of this.^[19,20]^ Therefore, the authors believe that future studies should develop an observation method suitable for diffuse gastric cancer tumor outgrowth, rather than relying only on the traditional HE staining observation. For example, explore whether hypo-differentiated clusters could be considered as a type of tumor budding? The study by Ana et al concluded that discrete differences in morphology and expression of epithelialmesenchymal transition (EMT) -related markers between tumor budding and poorly differentiated clusters indicate that they represent different manifestations of partial EMT.^[21]^ Thus, further investigation of the phenomenon of tumor budding in diffuse gastric cancer is needed, and it is essential to establish a conclusion on the universality of tumor budding across different types of gastric cancer. This requires further exploration of the intrinsic nature of tumor budding based on previous research.

### 3.2. Tumour budding in colorectal cancer

Colorectal cancer (CRC) is a prevalent malignant tumor of the digestive tract, with an incidence rate second only to gastric cancer. Its incidence and mortality rates rank fourth and sixth, respectively, among all malignancies.^[22]^ The correlation between tumor budding and CRC was first studied among malignant tumors, and hence, the relevant research in this area is relatively well-developed. The phenomenon of tumor budding in CRC has been extensively investigated, and numerous studies have confirmed its predictive value for poor prognosis and early recurrence in CRC.^[23]^ Specifically, high-grade tumor budding is associated with a worse prognosis and shorter overall survival (OS) compared to low-grade tumor budding. Recently, scholars have also discovered that the combination of tumor budding and the form of tumor growth holds significance in assessing the prognosis of CRC, and that tumor budding serves as an independent risk factor for CRC prognosis.^[24]^ Furthermore, studies have indicated that tumor budding is informative for the treatment of early colon cancer at T1N0M0 stage. In patients with tumor budding, it can significantly impact their disease-free survival.^[25]^ Therefore, the tumor budding technique can be incorporated as a routine component of the pathological examination following microscopic treatment of early colon cancer. For patients with indications of high-grade tumor budding, close monitoring of their prognosis or direct progression to further surgical treatment is recommended to prevent metastasis or recurrence.

## 4. Biological behavior of tumor budding

### 4.1. Correlation between tumor budding and invasiveness

Tumor budding (TB) is defined as the shedding of single tumor cells or small clusters (≤4 cells) from the margins of tumor tissue. This microscopic manifestation is widely recognized as indicative of the invasiveness and metastatic potential of malignant tumors. TB is a dynamic process, as Bronsert study demonstrated, where initial finger-like projections form at the tumor edge.^[26]^ These projections consist of numerous tumor cells that eventually separate into individual cells or small clusters. TB can be considered an excellent marker for differentiating between benign and malignant tumors, as malignant tumors tend to exhibit visible TB, whereas benign tumors usually do not. The European Society for Medical Oncology consensus guidelines recommend resecting colorectal polyps that show TB, as they are indicative of malignant transformation and require appropriate management.^[27]^ TB has been observed in various sites of malignancy, with tumor cells often appearing as strips or thin threads when shed from the primary tumor tissue. This shedding process is considered the first step in the metastatic cascade of malignant tumors.^[7]^ Dislodged tumor cells can spread to distant sites through blood vessels or lymphatic vessels, or they can be directly implanted in a metastatic location near the primary tumor.

### 4.2. Tumour budding and epithelialmesenchymal transition

Epithelial-mesenchymal transition (EMT) is a process in which epithelial cells transform into mesenchymal cells. This process plays a crucial role in embryonic development, tissue repair, and the metastasis of malignant tumors.^[28]^ EMT is a dynamic rather than static phenomenon. During EMT, adhesion between epithelial tissues decreases, and the cells gradually acquire mesenchymal-like biological characteristics, such as invasiveness and metastasis. This transition is characterized by a significant downregulation of E-cadherin expression and an upregulation of N-cadherin and vimentin, leading to a morphological shift from normal epithelial cells to polygonal or pebble-shaped cells.^[29]^ This change results in a marked decrease in cell polarity, a decline in cell-to-cell adhesion, and the gradual disappearance of cell-to-cell connections, paving the way for the subsequent steps of tumor metastasis. Once shed from the primary tumor, tumor cells disseminate and metastasize to distant sites via blood vessels or lymphatic vessels. However, the metastatic journey is fraught with challenges. Most tumor cells are eliminated by immune surveillance, such as macrophages in blood vessels and lymphatic vessels, and only a few reach the target site. Upon arrival, the tumor cells undergo a reverse process termed mesenchymal-epithelial transition (MET), during which they reexpress E-cadherin, regain polarity, and restore cell-to-cell adhesion. This allows the tumor cells to adhere to normal tissue cells in the target area, stabilize at the site, proliferate, and differentiate, ultimately forming metastatic foci of the malignancy.^[30]^

Tumor metastasis and recurrence at the macroscopic level have been observed to be intimately linked to the processes of tumor budding, as well as EMT and MET at the molecular and cellular level. The dynamic activity occurring at the cellular and molecular level results in alterations in tumor cell adhesion, ultimately facilitating metastasis and completing the process of malignant tumor dissemination. These findings underscore the importance of understanding the intricate interplay between these cellular and molecular processes in driving tumor progression and recurrence.

### 4.3. Recent advances in research on tumor budding

A study by Susan et al showed that tumor budding could be a prognostic biomarker in biopsies and resections of neoadjuvant-treated rectal adenocarcinoma.^[31]^ Pericytes were identified as an important component of the tumor microenvironment. The separation of pericytes from endothelial cells facilitates the invasion of in situ tumor cells into the circulating bloodstream and favors local capillary basement membrane enzymolysis and endothelial cell proliferation and budding, thus promoting tumor angiogenesis and metastasis.^[32]^ Liang et al showed that elevated levels of KRT17TB were negatively correlated with the grading of TB. This suggests that KRT17TB can inhibit tumor budding to a certain extent.^[33]^ Ugur has developed a method to predict colorectal cancer tumor budding based on preoperative hemoglobin, albumin, lymphocytes and platelets. However, the method was only moderately discriminating in moderate to high tumor budding (*P* = .032) with a sensitivity of 70.89% and a specificity of 48.39%.^[34]^

## 5. Discussion of tumor budding and new targets in tumor therapy

### 5.1. Tumour budding and LncRNAs

Long noncoding RNAs (lncRNAs), which are longer than 200 nucleotides and do not encode protein products, have emerged as key regulators in various biological processes, including normal growth and development, as well as tumorigenesis and progression.^[35]^ In recent years, several Chinese scholars have discovered a novel lncRNA, termed Lnc-CTSLP4, that may play a pivotal role in EMT and tumor budding.^[36]^ When cells were transfected with a vector overexpressing this lncRNA, they exhibited significantly reduced invasiveness compared to control cells. Furthermore, Lnc-CTSLP4 was found to upregulate E-cadherin expression and downregulate N-cadherin levels in these cells, leading to increased cell-cell adhesion at the leading edge of the tumor infiltrate. These findings suggest that Lnc-CTSLP4 may inhibit tumor budding by promoting cell-cell adhesion, thereby influencing tumor progression and metastasis.

### 5.2. Relationship between tumor budding and CDH17

Cadherin 17 (CDH17), a member of the 7D cadherin superfamily, plays a crucial role in intercellular adhesion.^[37]^ Recent studies have reported that CDH17 is overexpressed in gastric cancer tissues and is associated with metastasis and poor prognosis in gastric cancer patients.^[38]^ These findings highlight the potential importance of CDH17 in gastric cancer progression and suggest that it may serve as a therapeutic target or prognostic indicator in this disease.

### 5.3. Tumour budding and miRNAs

MicroRNAs (miRNAs), a class of noncoding RNAs approximately 18 to 25 nucleotides in length, play pivotal roles in regulating gene expression by binding to specific 3’ untranslated regions (UTRs) of target mRNAs, thereby negatively regulating translation and protein synthesis.^[39]^ Recent research has demonstrated that these regulatory effects are often due to incomplete complementarity between the miRNA and its target mRNA, although complete complementarity can lead to mRNA degradation.^[40]^ Studies by Aaron and his team have identified nearly 100 miRNAs with dysregulated expression in colorectal cancer samples, suggesting that these miRNAs may be closely related to tumorigenesis, differentiation, and metastasis.^[41]^ Among these, miRNA-148a and miRNA-625-3p have garnered significant attention from scholars due to their potential roles in regulating EMT through modulation of E-cadherin, N-cadherin, fibronectin, and other proteins, which may promote tumor budding.^[42]^ Therefore, further investigation of these miRNAs may reveal novel therapeutic targets for the treatment of cancer.

### 5.4. Tumour budding and exosomes

Exosomes are vesicle-like structures with a diameter of approximately 40 nanometers that contain a variety of biologically active substances, including various RNAs and proteins. They are believed to originate primarily from the formation of multivesicular bodies through the invagination of intracellular lysosomal particles, which are subsequently released into the extracellular matrix following fusion of the outer membrane of the multivesicular body with the cell membrane.^[43]^ Exosomes are abundant in various body fluids and were initially thought to serve as a mechanism for the removal of degraded and unwanted cellular components. However, recent research has uncovered their critical roles in intercellular information transfer, tumor immune responses, and tumor cell migration and invasion.^[44,45]^

Several studies have demonstrated a close relationship between exosomes and the epithelial-to-mesenchymal transition (EMT) phenomenon. For instance, co-culturing nasopharyngeal carcinoma cell lines with exosomes isolated from similar cell lines results in an increase in N-cadherin expression and a downregulation of E-cadherin expression, suggesting that exosomes can induce EMT in nasopharyngeal carcinoma cells and promote tumor metastasis.^[46]^ Additionally, miR-23a present in exosomes regulates E-cadherin expression in lung cancer cells, prolonging the EMT process through the TGF-β pathway and thereby promoting the metastasis of lung cancer cells.^[47]^ These findings highlight the intimate connection between exosomes and EMT. Given the intertwined relationship between EMT and tumor budding, it is reasonable to hypothesize that exosomes also have a close relationship with tumor budding. As the effect of exosomes on tumor budding has been observed in various tumors, further research into the role of exosomes in gastrointestinal malignancies is warranted. By precisely inhibiting the production and dissemination of relevant exosomes, it may be possible to inhibit the occurrence of tumor budding at its source, thereby prolonging patient survival.

## 6. Summary

The concept of tumor budding has been proposed for over 70 years, and with the continuous advancement of science and technology, scholars’ understanding of this phenomenon has gradually deepened. In the future, the study of tumor budding is expected to become more comprehensive, leading to a more unified understanding of this feature. Additionally, the investigation of micro-areas will become more thorough, facilitating the discovery of novel therapeutic targets that can effectively address tumor infiltration, metastasis, and poor prognosis. This has the potential to profoundly impact the treatment of malignant tumors, including gastric cancer, as well as the assessment of prognosis. In future studies, the ultrastructure of tumor budding could be explored to enable better identification of tumor budding features in diffuse gastric cancer sites. At the same time, tumor budding in gastric and colorectal cancers should be combined with other tumour microenvironmental and serological indicators, and artificial intelligence should be used to predict the impact of tumor budding on postoperative recurrence and prognosis in gastric and colorectal cancers.

## Acknowledgments

We would like to thank the researchers and study participants for their contributions.

## Author contributions

**Project administration:** Hongyan Li.

**Writing – original draft:** Hao Zhang, Dongyun Gou, Haiyang Wang.

**Writing – review & editing:** Xianyu Meng, Yu Huang.
